# Transcriptome Analysis for Identification of Genes Related to Gonad Differentiation, Growth, Immune Response and Marker Discovery in The Turbot (*Scophthalmus maximus*)

**DOI:** 10.1371/journal.pone.0149414

**Published:** 2016-02-29

**Authors:** Deyou Ma, Aijun Ma, Zhihui Huang, Guangning Wang, Ting Wang, Dandan Xia, Benhe Ma

**Affiliations:** 1 Yellow Sea Fisheries Research Institute, Chinese Academy of Fishery Sciences, Key Laboratory of Sustainable Development of Marine Fisheries, Ministry of Agriculture, Qingdao Key Laboratory for Marine Fish Breeding and Biotechnology, Qingdao, 266071, China; 2 Laboratory for Marine Biology and Biotechnology, Qingdao National Laboratory for Marine Science and Technology, Qingdao, 266071, China; 3 Dalian Ocean University, Dalian, 116023, China; Institute of Oceanology, Chinese Academy of Sciences, CHINA

## Abstract

**Background:**

Turbot *Scophthalmus maximus* is an economically important species extensively aquacultured in China. The genetic selection program is necessary and urgent for the sustainable development of this industry, requiring more and more genome background knowledge. Transcriptome sequencing is an excellent alternative way to identify transcripts involved in specific biological processes and exploit a considerable quantity of molecular makers when no genome sequences are available. In this study, a comprehensive transcript dataset for major tissues of *S*. *maximus* was produced on basis of an Illumina platform.

**Results:**

Total RNA was isolated from liver, spleen, kidney, cerebrum, gonad (testis and ovary) and muscle. Equal quantities of RNA from each type of tissues were pooled to construct two cDNA libraries (male and female). Using the Illumina paired-end sequencing technology, nearly 44.22 million clean reads in length of 100 bp were generated and then assembled into 106,643 contigs, of which 71,107 were named unigenes with an average length of 892 bp after the elimination of redundancies. Of these, 24,052 unigenes (33.83% of the total) were successfully annotated. GO, KEGG pathway mapping and COG analysis were performed to predict potential genes and their functions. Based on our sequence analysis and published documents, many candidate genes with fundamental roles in sex determination and gonad differentiation (*dmrt1*), growth (*ghrh*, *myf5*, *prl*/*prlr*) and immune response (*TLR1*/*TLR21*/*TLR22*, *IL-15*/*IL-34*), were identified for the first time in this species. In addition, a large number of credible genetic markers, including 21,192 SSRs and 8,642 SNPs, were identified in the present dataset.

**Conclusion:**

This informative transcriptome provides valuable new data to increase genomic resources of *Scophthalmus maximus*. The future studies of corresponding gene functions will be very useful for the management of reproduction, growth and disease control in turbot aquaculture breeding programs. The molecular markers identified in this database will aid in genetic linkage analyses, mapping of quantitative trait loci, and acceleration of marker assisted selection programs.

## Introduction

Turbot (*Scophthamus maximus*) is an economically important flatfish widely farmed in Europe and Asia. The intensive culture of turbot has been promoted in the past few years because of great economic value. Turbot production boosted during the last decade in China, which has become the world’s largest turbot-producing nation reported by FAO in 2010. However, inbreeding and intensive culture has brought about multiple negative effects on turbot industry. Enormous economic losses resulted from the slow growth and disease outbreaks of fish [1]. Thus, genetic breeding programs in this species are accessible to be carried out. Currently, the main targets of genetic improvement in turbot are controlling sex ratio, increasing growth rate and enhancing disease resistance [2].

Several economic traits are related to sex in aquaculture species. Sexual dimorphism has been observed in growth rate, time and age of maturation, body shape and carcass composition [3]. Turbot exactly exhibits the significantly sexual dimorphism for growth rate in favor of females among aquaculture species [4]. Producing all-female stocks seems promising to increase biomass of turbot for acquiring more interests. Therefore, controlling sex ratio is one of the major targets of genetic improvement in turbot. Understanding the process of gonadal development can offer a powerful support in the control of sex ratios in finfish aquaculture. An undifferentiated bipotential gonad of fish develops into either a testis or an ovary depending on sex determining genes [5], and external factors such as temperature or pH can directly influence gonadal development and then affect sex ratio in some fish [6], while the mechanisms of sex determination and gonad differentiation in turbot are not conclusive [7,8], due to the insufficient genomic information, and consequently need to be further explored. Growth rate from hatching to commercial size is a primary trait of interest in selection programs of most economic fish and has an intrinsic link with productivity and profitability of aquaculture enterprises. Growth of vertebrates (including fish) is primarily controlled by the GH-insulin-like growth factor-I (IGF-I) axis [9]. There are few studies on growth-related genes and corresponding regulation network in turbot. Intensive culture conditions in fish farms aggravate the risk of pathogen infection and the consequent losses of benefit associated with disease outbreaks. Reduction of disease occurrence is a major concern for turbot aquaculture [10]. Obtaining resistant broodstock is a fascinating solution to control diseases in front of the economic cost of vaccines, treatments and the possible generation of resistances against antibiotics. The necessary preparation is a comprehensive understanding of the immune system in economic fish species [11], particularly in turbot. During the last few years, a large number of expressed sequence tags (ESTs) from the turbots challenged with the most common different pathogens [12] have pooled into a database relevant to immune response. Despite recent increases in the number of gene sequences for turbot, the available genomic resources are inadequate yet to offer an extensive detection on candidate genes in control of economic traits and the corresponding regulatory pathways in this species.

Transcriptome containing most protein coding genes is a small but essential part of the genome. Sequencing the transcriptome is an attractive alternative for gene discovery in species whose genome is still not available, especially to economic fish. Newly-developed high-throughput sequencing technologies can produce huge transcriptomic data of non-model organisms with low cost and high efficiency [13]. More than 2000 differentially expressed sex-biased genes and several sex-related biological pathways were firstly found in flounder (*Paralichthys olivaceus)* based on an Illumina platform [14]. An overall database from multiple tissues of turbots was reported using a combined strategy involving Sanger and 454 pyrosequencing, in which important genes related to reproduction and disease control were discovered [15]. There are few reports on a whole transcript profile of turbot that has become a highly appreciated aquaculture species since its introduction to China.

The selection on basis of molecular markers including simple sequence repeats (SSRs) and single nucleotide polymorphisms (SNPs) is another approach to improve the aquaculture production in commercially important fish species [16]. Hundreds of SSRs have been developed and validated in turbot [17], many of which have been already used for genetic linkage mapping [18,19]. Several QTLs significantly associated with sex-determination [20], growth [21] and resistance to pathogens [2,22] were identified through a genome scan using the genetic map in turbot, which implies the existence of genetic factors underlying these characters and supports their application in genetic breeding strategies. Massive molecular markers have been exploited from extensive transcriptomic sequence data with the advances of sequencing technologies in a variety of economic species [23,24]. Hundreds of true SNPs were detected using 454-pyrosequencing method in turbot and most SNP-containing genes were related to immune response and gonad differentiation processes, which could be chosen as candidates to discover the relationship between functional changes and phenotypic changes [25].

In this study, the transcriptome of pooled multiple tissues from one male and one female turbots was characterized using an Illumina sequencing platform to maximize the chance of presenting as many transcripts as possible, respectively. The turbot genomic database is enriched by unigenes that were *de novo* assembled and annotated through strict bioinformatic analysis in this study. Many important genes involved in sex-control, growth and immune response were identified. Furthermore, abundant markers including SSRs located within coding regions and SNPs detected amongst deep coverage sequence regions reads were developed. Briefly, the transcriptome offers an invaluable data for further genomic research of flatfishes, as well as to discover new markers potentially useful in promoting turbot molecular breeding progress.

## Materials and Methods

### Ethics statement

The animals used in the present study were artificially cultivated, and all experimental treatments are implemented according to the recommendations in the Guide for the Care and Use of Laboratory Animals of the National Institutes of Health. The study protocol was approved by the Experimental Animal Ethics Committee, Yellow Sea Fisheries Research Institute, Chinese Academy of Fishery Sciences, China.

### Sample preparation

Two adult turbots of three years old including one male and one female (total length: ♀, 40.7 cm; ♂, 31.6 cm) used for transcriptome sequencing were purchased from Tian-yuan Fisheries Co. Ltd., Yantai, China. The fish samples were acclimated in the laboratory for one week before the experiment treatment. A variety of tissues including liver, spleen, kidney (inclusive of head kidney), cerebrum, gonads after breeding season and skeletal muscle were dissected from the two samples after euthanasia by immersion in MS-222 buffered solution (3 g/L) on ice. All fresh tissues were frozen in liquid nitrogen immediately and stored at -80°C until RNA extraction within 2 weeks.

### RNA isolation, cDNA library construction and Illumina deep sequencing

Total RNA was extracted from each tissue sample using Trizol Reagent (Invitrogen, CA, USA). After checking RNA purity and concentration, the integrity of RNA samples was assessed using the RNA 6000 Pico LabChip with a Bioanalyzer 2100 (Agilent Technologies, CA, USA). mRNA was purified from total RNA that was predigested at 37°C for 1 h using DNase I using Micropoly(A) PuristTM mRNA purification kit (Ambion USA). The eligible mRNA samples (RIN values≥8) from one turbot individual were pooled in equal amounts to generate one mixed sample. A total amount of 10 μg mRNA per sample was used as input material for preparing one separate Illumina sequencing libraries.

The method of double cDNA synthesis was modified on basis of the published method [26]. Briefly, first strand cDNA was synthesized using GsuI-oligo(dT) and Superscript II reverse transcriptase (Invitrogen, USA). Subsequently, the first strand was lysed from biotin-attached mRNA/cDNA that had been picked out by DynalM280 magnetic bead (Invitrogen) through recognition of biotin linked to mRNA 5’ cap structure. Second strand cDNA synthesis was subsequently performed using Ex Taq polymerase (Takara). The polyA ends and 5’ adaptors were eventually removed by GsuI enzyme.

The double cDNA was cleaved into fragments (300~500 bp) using Fisher ultrasound equipment and then purified by Ampure beads (Agencourt, USA). The generation and amplification of cDNA libraries were carried out by Illumina TruSeq RNA Sample Preparation Kit and TruSeq PE Cluster Kit (Illumina, San Diego, USA) following manufacturer’s recommendations, respectively. Finally, the libraries were sequenced on an Illumina Hiseq 2000 platform in the Chinese National Human Genome Center (Shanghai) and paired-end reads with approximate length of 100 bp were generated.

### Bioinformatic analysis

#### Quality control

Raw reads were produced through base calling and stored in fastq format. The raw data became clean after filteration by removing the adapter sequences, reads with unkown nucleotides (N) more than 10% and low quality sequences (base quality score<Q20). Clean data with high quality were the basis of following analyses.

#### Transcriptome assembly and gene annotation

*De novo* assembly of clean reads was carried out using Trinity software (http://trinityrnaseq.sf.net). Trinity was usually consisted of three independent software modules: Inchworm, Chrysalis and Butterfly. Using this software, sequencing data were partitioned into many single de Bruijn graphs (each represented transcriptional complexity for a given gene). Full-length splicing isoforms and transcripts from paralogous genes were obtained after the independent processing of graphs. At this period, the k-mer value was set to 25. The longest transcript from the same component was only preserved as a contig for excluding the interference from alternative splicing of transcripts. The assembled sequences were defined as unigenes.

The prediction of unigenes was performed by mapping to protein-coding sequences using GetORF of EMBOSS [27]. The predicted protein-coding sequences were annotated to the NCBI non-redundant (Nr) protein database and UniProtKB database, using BLASTp with algorithm with an E-value threshold of 1e-5. Gene encoding protein domains were identified by searching against Swiss-Prot and TrEMBL databases through BLASTp program. GO function for all unigenes was classified using Gene2go of GoPipe program [28]. KEGG (Kyoto Encyclopedia of Genes and Genomes) metabolic pathway annotation and COG (Clusters of Orthologous Groups) classification of unigenes were determined by searching against KEGG database and COG database using BLAST algorithm, respectively.

#### Expression abundance and GO enrichment analysis

Differential expression of unigenes in the two turbot libraries was analyzed using the MA-plot-based method with Random Sampling model (MARS) in DEGseq R package [29]. *P* value was adjusted by means of q value. Q value<0.001&|log2 (fold change)| >1 was set as the threshold for significantly differential expression. GO enrichment analysis of the differentially expressed genes (DEGs) was performed using a hyper geometric distribution test. GO term with false discovery rate (FDR)≤0.01 was defined as the term of significantly enriching DEGs.

#### Markers detection

Molecular markers, including SSRs and SNPs, were detected after mapping all clean reads to the assembled transcripts. The set of unique sequences was searched for SSR markers using MISA (http://pgrc.ipk-gatersleben.de/misa/misa.html). The minimum repeat number used for this search was eight for dinucleotide, five for tri-, four for tetra- and three for penta- and hexanucleotide microsatellites. SSR-containing ESTs were identified as candidates for marker development if they presented enough flanking sequences on either side of the repeats for primer design using Primer 3 (http://primer3.sourceforge.net/releases.php). Putative SNP detection was performed with SOAPsnp software. For identification of potential SNPs, various parameters such as base quality score and read depth were optimized. The following criteria were selected as the final SNP sets: read depth of four and the minimum variant frequency of two, variations compared to the consensus sequence were counted as SNPs. Furthermore, they were considered statistically significant at FDR/tested p-value<0.1.

### SSR validation and polymorphism evaluation

Genomic DNA was isolated from 90 randomly selected turbots using TIANamp marine animals DNA kit (TIANGEN, Beijing, China) according to the protocols. The integrity of DNA samples was checked using 1% agarose gel electrophoresis and their purity and concentration were assessed by Nanodrop 1000. All samples with the final concentration of 40 ng/μl were reserved at -20°C for upcoming analysis. The annealing temperature of primers was initially tested for amplification using a pool DNA samples. PCR amplifications were carried out using Master-cycler gradient thermal cycler (Eppendorf) in a final volume of 15 μl. Each reaction tube contains 1.4 μl 10× PCR buffer, 1.2 μl of dNTP (2.5 mM), 0.6 μl of each primer (10 μmol), and 1μl of genomic DNA (40 ng/ul), 0.2 μl of rTaq DNA polymerase (5 U/ ul,Takara), 10 μl of ddH_2_O. The PCR reaction program was: DNA denaturation at 95°C for 5 min; 30 cycles of 95°Cfor 45 s, 57~60°C for 50 s, 72°C for 50 s; and 72°C for 10 min as a final extension. Amplification products were resolved in 8% denaturing polyacrylamide gel, visualized by silver-staining to determine allele sizes using a 50-bp DNA ladder as a reference marker.

There were 17 pairs of SSR primers for the assessment of genetic diversity in turbot progenies. The number of alleles (*Na*), polymorphism information content (*PIC*), expected and observed heterozygosities (*He* and *Ho*, respectively) were calculated with the software PopGene32 (version 1.32).

### SNP validation

Genomic DNA of 96 individuals obtained from eight selected families was extracted from tail fins, using TIANamp marine animals DNA kit (TIANGEN, Beijing, China) following the manuscripts. DNA samples were prepared at -20°C with the concentration of 40 ng/μl. In total, 147 SNPs with the coverage ≥500 were chosen as candidates to validate the putative SNPs identified in transcripts, using high resolution melting (HRM) technology. Primers of high quality were designed using Primer premier 5.0 software and synthesized in Sangon (Shanghai, China), where the unlabeled oligonucleotides as the internal temperature controls for genotyping by amplicon melting [30] were produced. HRM genotyping was performed on LightScanner device using primers with distinct and single amplified products and LC Green Plus dye. The genotyping data were analyzed by PopGene32 (version 1.31).

## Results and Discussion

### Illumina sequencing, reads assembly and gene annotation

Total RNA was isolated from multiple tissues of two adult turbots (one male and one female), including liver, spleen, kidney, cerebrum, gonad and skeletal muscle, for achieving a full-scale *S*. *maximus* transcriptome. RNA samples were pooled with equal quantities to construct two cDNA libraries and sequenced by two Illumina platforms, respectively. This mixing strategy was commonly reported in some similar studies [23,31,32]. In total, 44,219,773 clean reads of 100 bp in length were received from the two libraries of one male and one female turbot samples after trimming adaptors and low-quality sequences (Table 1). The remaining reads were assembled into 106,643 contigs, of which 71,107 were left as unigenes by eliminating redundancies. As indicated in Table 1, the length distribution of all unigenes was 201~17407 bp with a mean length of 892 bp. The final assembled sequences and detailed gene annotations were presented in S1 File and S1 Table, respectively.

**Table 1:** Summary of Illumina transcriptome, assembly and annotation for *Scophthalmus maximus*.

Raw results (after trimming)		Assembly results		Annotation results	
Clean bases (G)	8.844	Contigs	106,643	Nr and Swiss-Prot annotations	24052
Read pairs	44,219,773	Unigenes (after eliminating redundancy)	71,107	COG hits	37058
Read length (bp)	100	Min-Max length of unigenes	201–17,407	GO mapped	16540
		Average length of unigenes	892	KEGG hits	11938

### Unigene annotation

**Fig 1:**
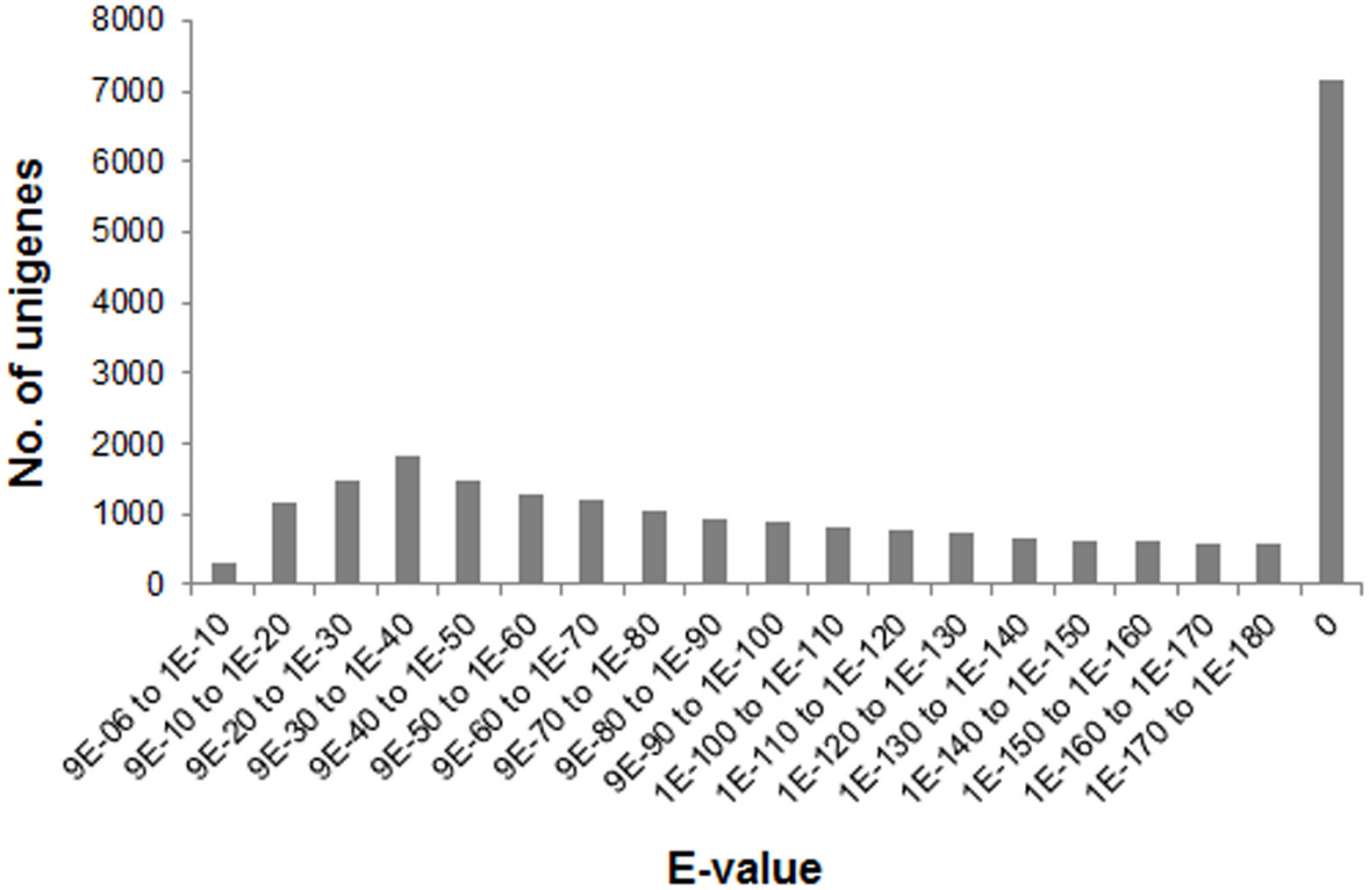
*E*-value distribution of *S*. *maximus* transcriptome unigenes matched to Nr database.

The alignment of non-redundant unigenes was performed with public Nr and Swiss-Prot databases to estimate their putative function. Totally 24,052 unigenes, which took up an approximate proportion of 33.83%, were annotated to the know sequence databases with significant blast scores. As shown in Fig 1, more than half of annotated sequences (50.25%, 12,087) had an *E*-value from 9E-10 to 1E-110, while 29.38% (7,162) with the *E*-value to be zero. Nearly two-third (66.17%) of *S*. *maximus* unigenes were not annotated to any sequences in the reference databases. The low annotation ratio seems unsurprising in non-model organisms without published genomes, especially aquaculture varieties [33–35]. Previous studies on transcriptome analyses indicate that unannotated sequences mainly represent transcripts of spanning only untranslated mRNA regions, chimeric sequences derived from assembly errors [36] and containing non-conserved protein regions [37]. Some may also be components of novel genes specific to this species, which are likely to be matched to certain genome sequences in the near future.

**Fig 2:**
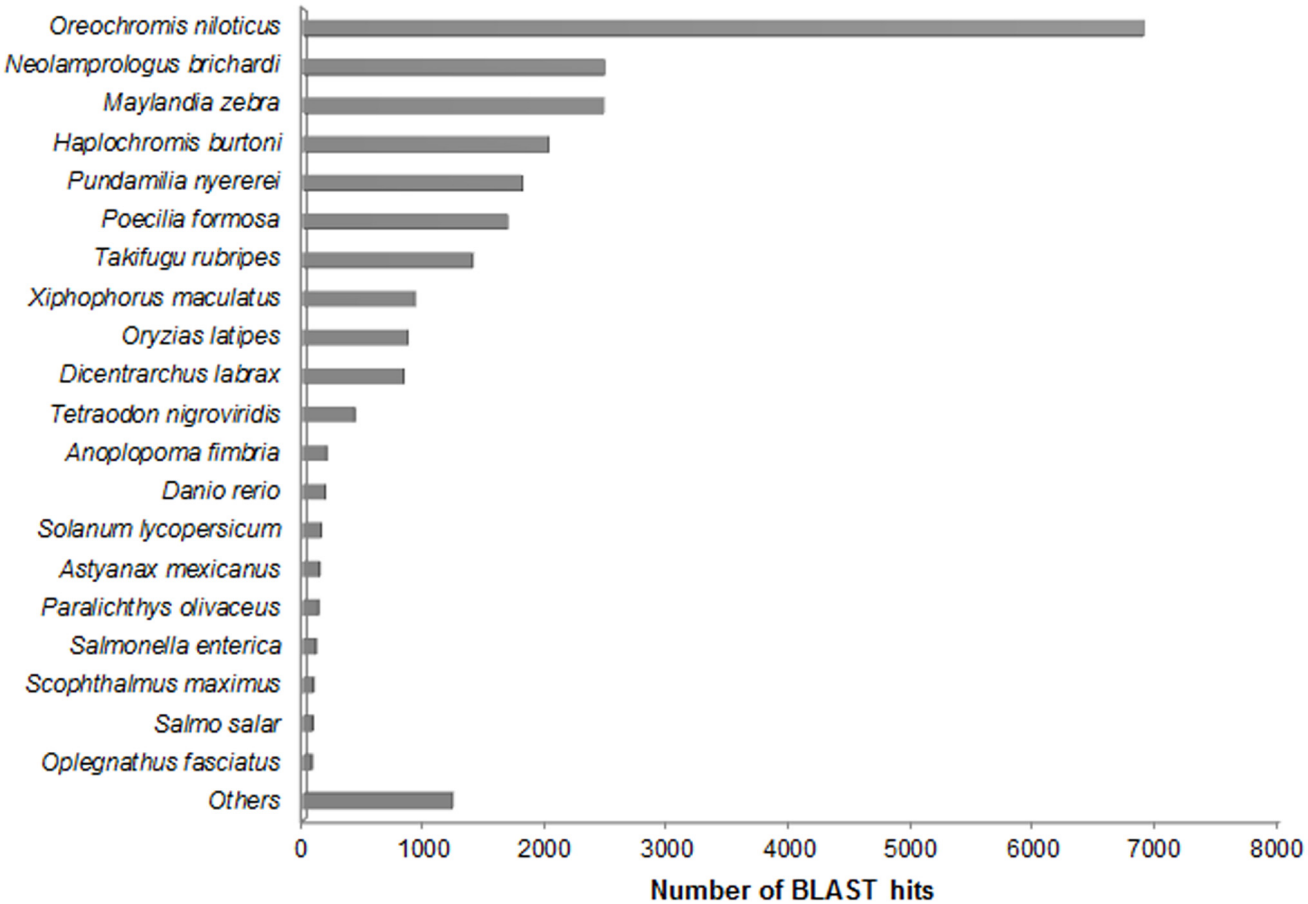
Top 20 hit species distribution based on BLASTp.

The result of main species distribution matched against Nr database (Fig 2) showed that 28.74% of the annotated unigenes shared similar sequences with *Oreochromis niloticus*, whose draft genome was published in 2014. The following species were *Neolamprologus brichardi* (10.29%), *Maylandia zebra* (10.27%), *Haplochromis burtoni* (8.38%), *Pundamilia nyererei* (7.48%), *Poecilia formosa* (6.98%), *Takifugu rubripes* (5.77%), *Xiphophorus maculatus* (3.80%), *Oryzias latipes* (3.56%), *Dicentrarchus labrax* (3.41%), *Tetraodon nigroviridis* (1.75%), and others (5.10%). As expected unigenes of turbot transcriptome matched well to proteins of other fish including species with reported genome. The ratio of unigene annotation to *S*. *maximus* was only 0.33%, which could be due to its few genomic sequences submitted in Genbank.

### Classification of COG, GO and KEGG

Sequences of assembled unigenes were also subjected to BLASTp searching against databases of COG, GO and KEGG. Summary of statistical results were shown in Table 1.

**Fig 3:**
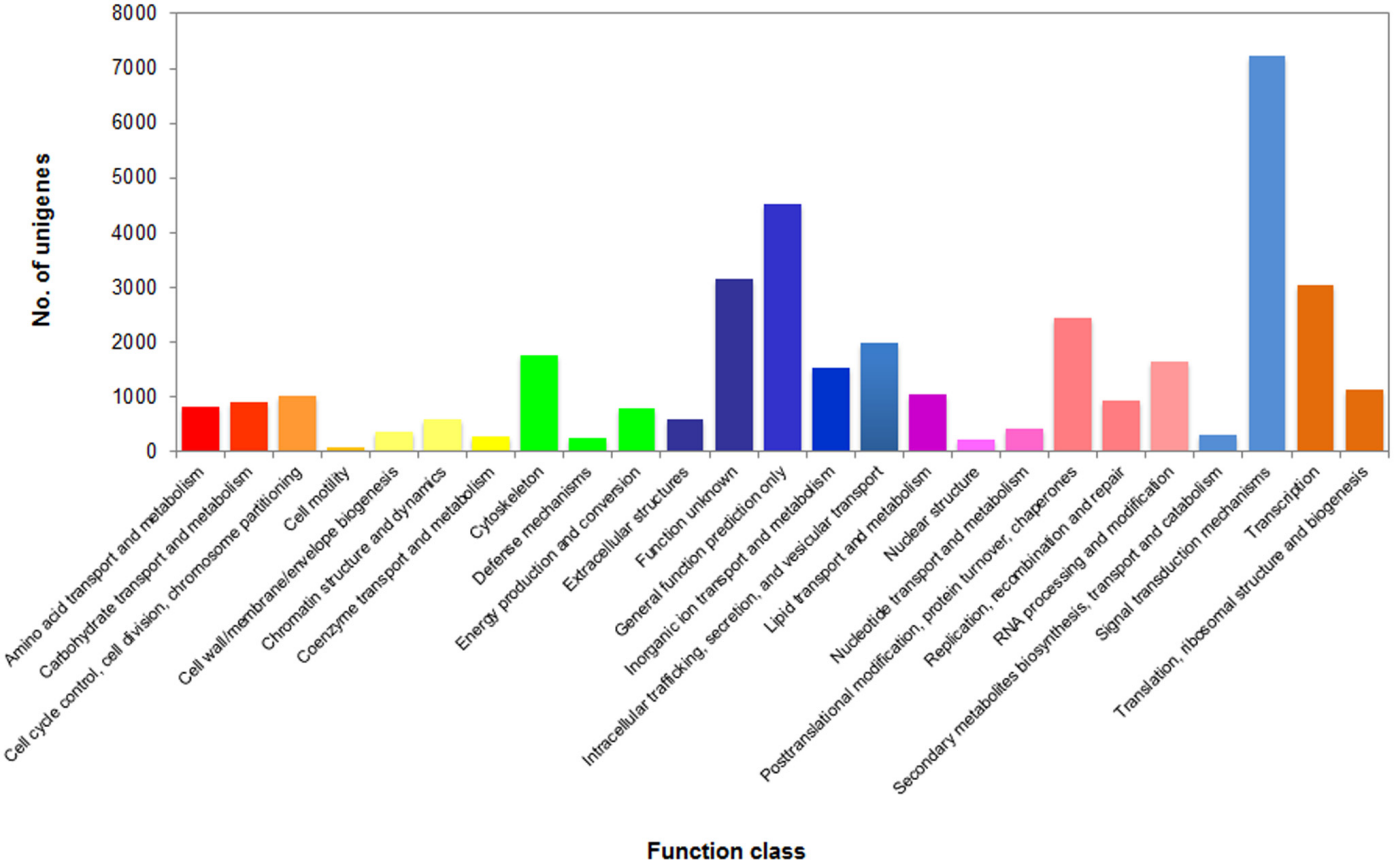
COG classification of putative proteins for *S*. *maximus* transcriptome.

COG database provides the classification of orthologous gene products. Unigene annotations of COG were selected for checking the completeness of our transcriptome library and the effectiveness of the annotation process. The possible functions of unigenes were predicted and classified by searching their predicted CDSs of unigenes against COG database (Fig 3). Possible functions of 37,058 unigenes were clustered into 25 COG categories, and the top five were ‘signal transduction mechanisms’ (7,236), ‘general function prediction only’ (4,521), ‘function unknown’ (3,143), ‘transcription’ (3,034), and ‘posttranslational modification, protein turnover, chaperone’ (2432), while the three smallest clusters were ‘defense mechanisms’ (242), ‘nuclear structure’ (228) and ‘cell motility’ (93).

**Fig 4:**
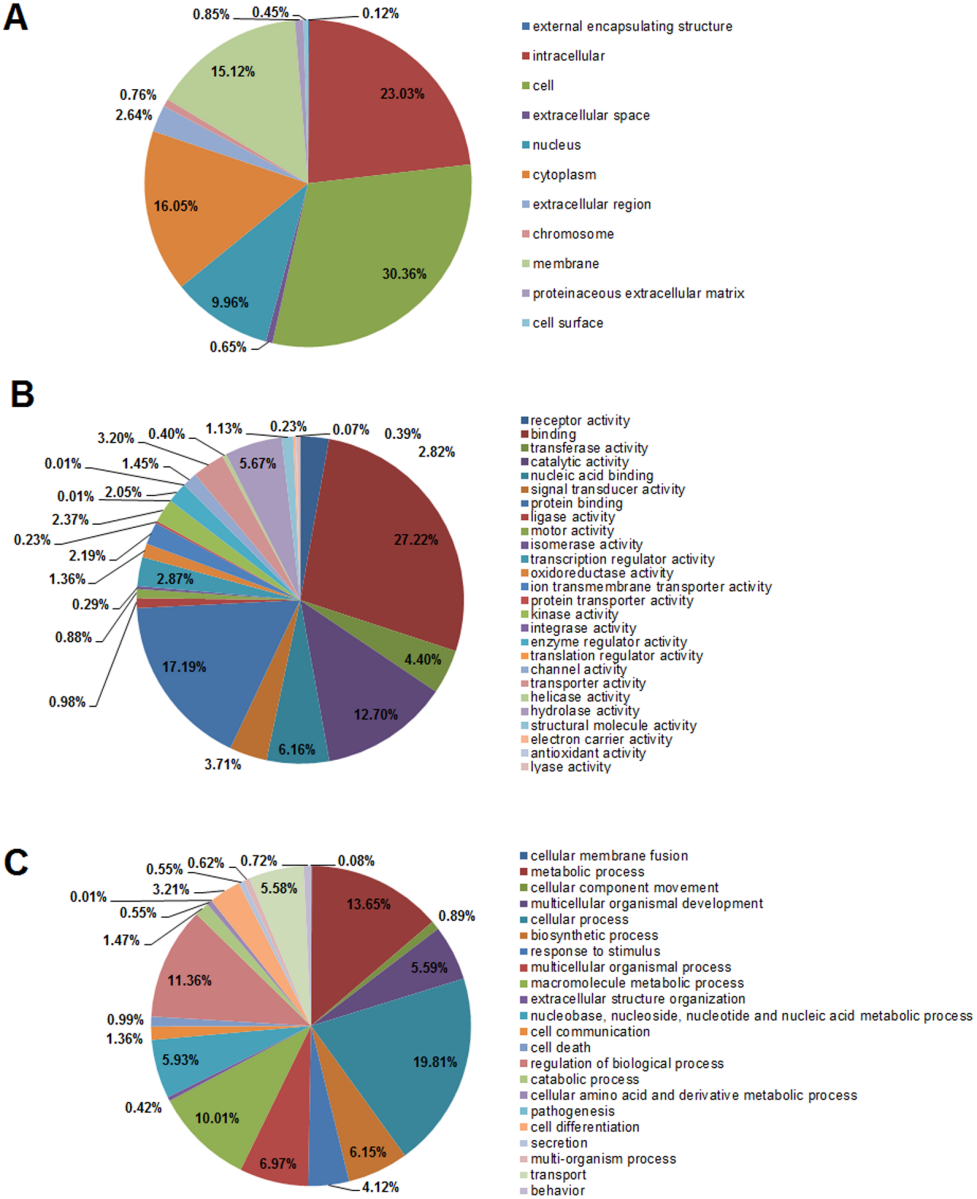
Distribution of Gene Ontology (GO) functional categories (level 2) of transcripts for *S*. *maximus*. (A) Cellular component; (B) Molecular function; (C) Biological process. Each annotated sequence is assigned at least one GO term. Numbers refer to percentage of assigned unigenes in each category.

Gene Ontology (GO) is an international classification system of standardizing gene function across species to comprehensively profile characteristics of genes, gene products and sequences [38]. In this study, 16,540 unigenes were categorized by GO analysis (Table 1). Second-level GO terms were used to classify the involvement terms of unigenes in three main categories (cellular component, molecular function and biological process) and each unigene was assigned to one or more GO term. In this study, 14,633 unigenes are involved in cellular component categories, among which, ‘cell’ (14,155 unigenes; 30.86%), ‘intracellular’ (10,740; 23.03%), ‘cytoplasm’ (7,486; 16.05%) and ‘membrane’ (7,050; 15.12%) comprised the largest proportion (Fig 4A). Further, 11462 unigenes are involved in 25 level-2 terms of molecular function category, and ‘binding’ (12,114; 27.22%), ‘protein binding’ (7,650; 17.19%), ‘catalytic activity’ (5,651; 12.70%) and ‘nucleic acid binding’ (2,743; 6.16%) were the most abundant (Fig 4B). Additionally, 13,676 unigenes are involved in various biological process categories, as shown in Fig 4C, the top four terms were ‘cellular process’ (10,468; 19.81%), ‘metabolic process’ (7,213; 13.65%), ‘regulation of biological process (6,003; 11.36%), and ‘macromolecule metabolic process’ (5,287; 10.01%). In summary, these GO terms cover a majority of the overall assignments in *S*. *maximus* transriptomic dataset. It is easily understood that genes encoding these functions may be well annotated in the database as a result of their conservation across different species.

**Fig 5:**
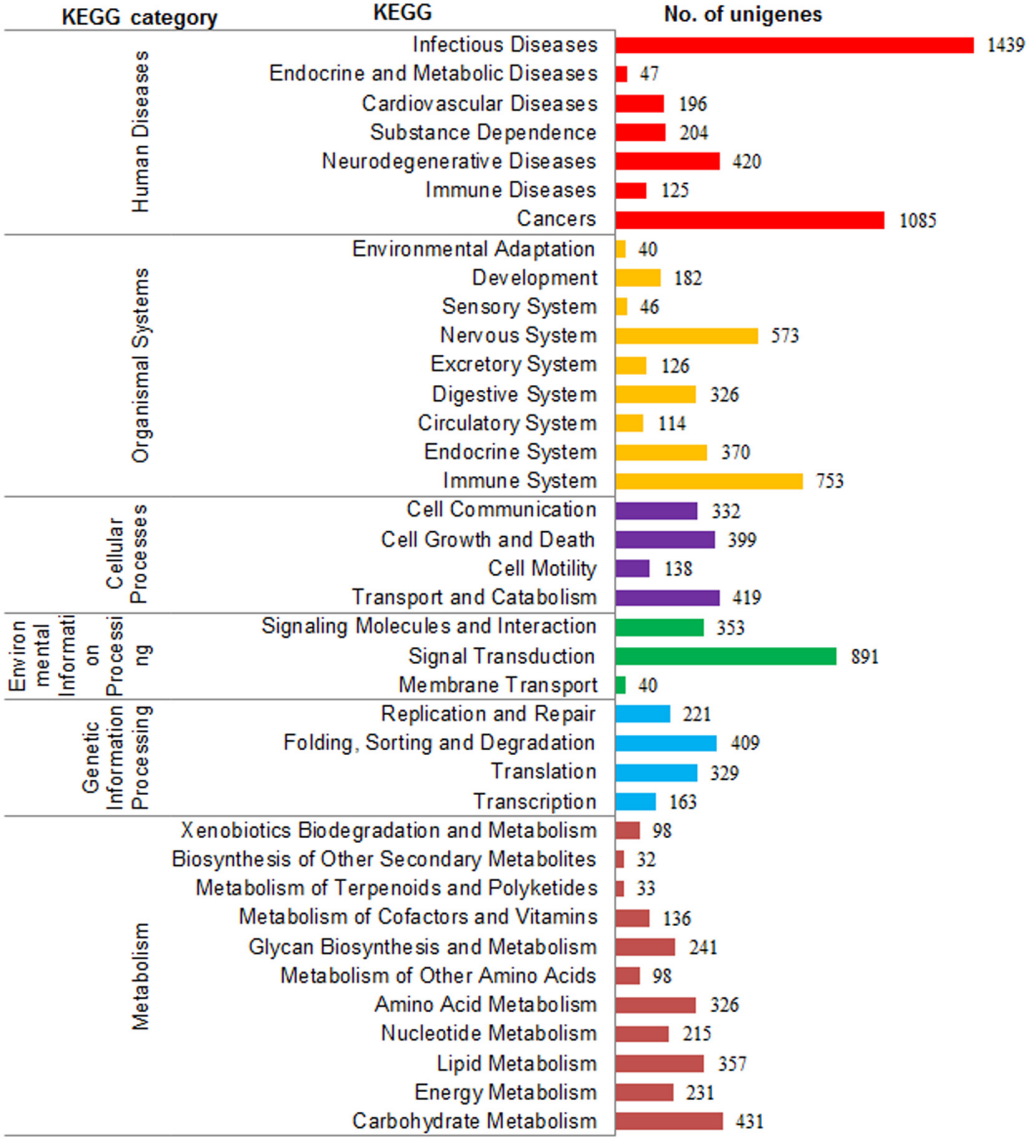
KEGG classification of non-redundant unigenes for *S*. *maximus* transcriptome.

KEGG pathway-based analysis facilitated to further study complicated metabolic pathways and biological behaviors of genes [39]. A total of 11,938 unigenes were consequently classified into specific pathways (Fig 5), among which most fell into ‘human diseases’ (3,516), ‘organism system’ (2,530), and ‘metabolism’ (2,198), followed by ‘cellular processes’ (1,288) and ‘environmental information processing’ (1,284), while least were assigned to ‘genetic information processing’ (1,122). Predominant subcategories of all the pathways were ‘infectious diseases’ (1439), ‘cancers’ (1085) and ‘signal transduction’. These annotations offer a valuable resource for investigating specific processes, functions, and pathways in flatfish research.

### Identification of genes related to sex determination, growth and immunity

#### Genes related to sex determination and gonad differentiation

As one of the most promising aquaculture species in Europe and China, turbot shows extreme differential growth rates between sexes. Compared with males, significantly faster growth rate and later sexual maturity of females make all-female population production desirable for turbot industry [40]. Several studies revealed the sex ratio of turbot is determined by major genetic sex-related factors [41] and limitedly influenced by environmental factors like temperature [42]. Genes involved in sex differentiation and gonad development play obvious roles in controlling sex ratio of this species. It seems reasonable that elucidating the mechanisms of sex determination and gonad differentiation is a prior goal to boost turbot production, but delayed due to insufficient known sex-related genes and explicit biological pathways in turbot.

In this study, more than 44 million clean reads were produced using Solexa technology and a large number of unigenes (71,107) were strictly annotated after de novo assembly. The selection criterion on sex-associated genes was mainly referred to the comprehensively gonadal transcriptome information of turbot [15] and olive flounder [14]. Table 2 shows 40 relevant genes including both well-known genes in SD of fish and novel genes identified for the first time in turbot. Our result shows some genes (*ar*, *mis*, *sox9*, *sox6*) proved to play roles in testicular development, consistent with the report of Ribas *et al* [15]. The *sox* genes encode an important family of transcription factors with highly conserved HMG domain [43], involved in a variety of developmental processes including sex determination and differentiation. Sox9 is an important member of SoxE group and attracts extensive attention, because *sox9* enables to lead male development even in the absence of *sex-determining region* of the Y chromosome (*sry*), and plays a critical role in the male sex-determining pathway as the downstream gene directly regulated by SRY in vertebrates [44,45]. Several annotated genes including *sox6b* (*sox6* homologue), *ar*, *hsp90α*, *dnali1* and *ropn1l*, are considered to be correlated with turbot testicular development according to the male-specific expression proofs in flounder [14]. The *dmrt* genes have attracted considerable interest recently because of their involvement in sex determination and differentiation among animal phyla. Dmrt1 with a highly conserved zinc finger-like DNA-binding (DM), is confirmed as the master regulator of male gonad differentiation [46], while its action mechanisms have yet to be elucidated in the turbot. Recently, *dmrt1* was validated in the Z chromosome of half-smooth tongue sole (*Cynoglossus semilaevis*) that belongs to the same order with turbot [47]. This finding will intrigue researchers to seek the more reliable proof through in-depth study of *drmt1* on the sex ratio to define the sex determination pattern, based on its firstly identified coding sequence of turbot in this study.

**Table 2:** Identified genes involved in sex determination and development in the present turbot transcriptome.

Gene Name	Annotation	Query Name	Length	Expression quantity (♂/♀)	Q-value	Trend
***dmtr1***	Doublesex and mab-3-related transcription factor 1	comp30078_c2_seq1	566	6.43	1.36E-10	up
*fshr*	Follicle stimulating hormone receptor II	comp34552_c0_seq1	3389	2.39	2.73E-70	up
*sox9*	SRY-box containing protein 9	comp36634_c7_seq1	1879	-0.20	0.47	
*sox6*	SRY-box containing protein 6-like	comp21968_c1_seq1	560	2.53	0.023	
*ar*	Androgen receptor	comp34574_c1_seq1	411	-0.51	0.69	
*gsf*	gonadal soma derived factor 1	comp38118_c0_seq1	2047	1.33	1.4E-160	up
*mnd1*	meiotic nuclear division protein 1 homolog	comp34969_c0_seq1	1598	-0.75	2.7E-07	
*mis*	Müllerian inihibiting substance	comp15802_c0_seq1	3092	0.22	0.477	
***hsp90α***	Cytosolic heat shock protein 90 alpha	comp18173_c0_seq1	294	0.07	1.00	
***dnali1***	Axonemal dynein light intermediate polypeptide 1	comp21140_c0_seq1	1007	8.30	5.61E-32	up
***ropn1l***	Ropporin-1-like protein-like isoform X2	comp28799_c0_seq1	1720	3.26	3.45E-19	up
*cyp19a*	P450 aromatase	comp20558_c0_seq1	2026	-7.64	5.1E-45	down
*zpc5*	Zona pellucida glycoprotein 5	comp20390_c0_seq1	1801	-12.65	0	down
*zar1*	Zygote arrest protein 1-like	comp20043_c0_seq1	1446	-12.54	0	down
*gtc*	Gonadotropin alpha	comp28377_c1_seq1	830	-11.45	0	down
*gdf9*	Growth differentiation factor 9	comp30868_c0_seq1	3680	-5.76	0	down
*star*	Steroidogenic acute regulatory protein	comp23962_c0_seq1	967	0.32	1	
*start-5*	StAR-related lipid transfer protein 5-like	comp35171_c2_seq1	5165	-1.50	0	down
*start-7*	StAR-related lipid transfer protein 7-like	comp37065_c0_seq1	3481	-2.00	0	down
*sox17*	SRY-box containing protein 17	comp26556_c1_seq1	266	1.07	0.48	
*sox19*	SRY-box containing protein 19	comp34381_c0_seq1	2961	-2.04	0	down
*foxl2*	Forkhead transcription factor L2	comp34087_c3_seq1	1401	-5.00	2.68E-46	down
***vtgr***	Vitellogenin receptor isoform 1	comp36664_c17_seq1	3808	-3.35	0	down
*zp4*	Zona pellucida sperm-binding protein 4-like	comp30721_c0_seq1	2238	-9.38	3.1E-122	down
*zp3*	Zona pellucida sperm-binding protein 3-like	comp29565_c0_seq1	1247	-13.66	0	down
*zp*	Zona pellucida sperm-binding protein	comp28111_c0_seq1	1390	-12.61	0	down
***fem1c***	Protein fem-1 homolog C-like	comp35674_c0_seq1	4180	-2.02	1.2E-151	down
***fzd3***	Frizzled-3-like	comp36698_c1_seq1	3514	-1.84	5.9E-209	down
***42sp43***	P43 5S RNA-binding protein-like	comp20349_c2_seq1	1515	-8.56	0	down
***gtf3a***	Transcription factor IIIA-like	comp17728_c0_seq1	1282	-14.12	0	down
***wee2***	Wee1-like protein kinase 2-like isoform X1	comp36348_c0_seq1	1904	-7.57	0	down
***ovca2***	Ovarian cancer-associated gene 2 protein homolog	comp31026_c0_seq1	1363	-5.28	2.2E-289	down
***ctss***	Cathepsin S-like	comp38095_c0_seq1	2038	-8.92	0	down
*vasa*	Vasa	comp36885_c2_seq1	2658	-0.21	0	
*dmrt2*	Doublesex and mab-3-related transcription factor 2	comp30078_c0_seq1	1113	-4.68	9.19E-07	down
*dmrt3*	Doublesex and mab-3-related transcription factor 3	comp29037_c2_seq1	1392	-1.55	0.00073	down
***dmrt5***	Doublesex and mab-3-related transcription factor 5	comp556746_c0_seq1	291	-1.51	0.519288	
*sox8a*	SRY-box containing protein 8a	comp36634_c2_seq1	2437	-1.54	2.35E-13	down
*shbg*	Sex hormone binding globulin	comp17530_c0_seq1	2212	-1.33	4.1E-62	down
*cyp1b*	Steroid 11-beta-hydroxylase	comp248709_c0_seq1	422	1.49	0.23	

Genes with Italics bold are novel.

Another group of identified genes in turbot is involved in ovarian development. The female-biased genes found in the present transcriptome, such as *cyp19a*, *zpc5*, *zar1*, *gtc5*, *gdf9*, *star*, *start-5/7*, and *gtc*, are well-established in the previous study [15]. Most of them are responsible for the synthesis of female hormones [48]. The transcripts of *sox17* and *sox19* genes were significantly upregulated in the differentiation of the ovary in sea bass *Dicentrarchus labrax* [49,50], implicating their roles in ovarian development of fish. *Foxl2* has been validated to be expressed exclusively in female, and joins in ovarian development as the encoding gene of *cyp19a* activating transcription factor [51]. The remaining genes are considered to be engaged in oogenesis (*wee2*), oocyte differentiation and development (*zp*, *42sp43*) and vitellogenesis (*vtgr*), justified by Fan et al. [14]. Moreover, *vasa* expressed in the germ cell were identified, which is involved in germ cell determination and development and plays an important role in formation of the primordial germ cell and migration to the germinal ridge [52]. The expression patterns of the above mentioned sex-biased genes support their roles in the turbot gonadal differentiation and development.

In this study, the differential expression of some sex-biased genes (including *dmrt2*, *dmrt3*, *sox8a*) was not consistent with their putative functions as reported in other teleosts. *Dmrt2-3* showed strong male-specific gonadal expression in adult testis of the medaka (*Oryzias latipes*) [53], while both male and female gonadal expression in developing germ cells of medaka [53], zebrafish (*Danio rerio*) [54] and swamp eel (*Monopterus albus*) [55]. The significantly male-biased expression pattern of *Sox8a* (*Sox8* homologue) in *Paramisgurnus dabryanus* [56], *Epinephelus coioides* [57] and *P*. *olivaceus* [14], suggesting this gene could be essential for differentiation of testis in fish. However, these genes could be assigned as female-biased class on the view of their expression favor in the female adult turbot transcriptome. Therefore, it is intriguing to probe into the diverse roles of these disputable genes in gonad differentiation as well as somite development [58].

#### Growth related genes

Growth rate of cultured fish from hatching to commercial size is one of the most important factors in the success of aquaculture. A variety of genes involved in regulating growth were identified from our sequence database (see Table 3), based on three principal search strategies: 1) associations between genes in the somatotropic axis and growth, 2) controlling growth at the muscle tissue level, 3) other candidate genes related to growth. A total of 60 assembled sequences, partial encoding regions of 17 genes or gene families, were verified in this study, which have been identified previously to have roles in growth of fish and other species.

**Table 3:** Genes of interest for growth and muscle development in *Scophthalmus maximus*.

	Gene Name	Annotation	Query Name	Length	Expression quantity (♂/♀)	Q-value	Trend
**Components of somatotropic axis**	*gh*	Growth hormone precursor	comp17564_c0_seq1	775	-10.66	4.01E-123	down
	*ghr*	Growth hormone receptor	comp34224_c0_seq1	1177	-0.78	0.04	
	*ghrh*	Growth hormone releasing hormone	comp374142_c0_seq1	325	2.66	0.10	
	*ghih*	Growth hormone inhibiting hormone-like	comp23996_c0_seq1	1004	0.32	0.59	
	*sstr*	Somatostatin receptor	comp24799_c1_seq1	1202	-1.13	0.0039	
	*igf*	Insulin-like growth factors	comp38041_c8_seq1	3754	-0.88	1.85E-45	
	*igfr*	IGF receptor	comp33325_c0_seq1	3008	-0.44	0.040	
	*igfbp*	IGF binding protein	comp32674_c4_seq1	1454	-1.43	4.03E-51	down
**Transforming growth factors**	*mstn*	Myostatin	comp20346_c1_seq1	2996	0.16	0.60	
	*myf5*	Myogenic regulatory factor 5	comp37586_c17_seq1	1393	-2.65	1.21E-34	down
**Others**	*fabp*	Fatty acid-binding protein	comp38300_c0_seq1	606	-4.13	0	down
	*htr*	5-Hydroxytryptamine receptor	comp26484_c1_seq1	1741	1.66	0.48	
	*prl*	Prolactin	comp38254_c0_seq1	747	-15.12	0	down
	*prlr*	Prolactin receptor	comp37125_c1_seq1	6331	-2.36	2.99E-188	down

Growth hormone-releasing hormone (GHRH), growth hormone inhibiting hormone (GHIH or somatostatin), growth hormone (GH), insulin-like growth factors (IGF-I and -II), and relevant carrier proteins and receptors, main components of the somatotropic axis, are widely accepted to play a critical role in regulating the formation of skeletal muscles in finfish [9]. As the main regulator of postnatal somatic growth, GH has been proved to play a vital role in stimulating anabolic processes such as cell division, skeletal growth and protein synthesis. Polymorphisms in the piscine *GH* gene have shown association with growth performance of *Salmo salar* [59] and *P*. *olivaceus* [60]. The biological actions of GH on target cells, including transmembrane signal transduction and subsequently transcriptional induction of many genes (e.g. *IGF-I*), are mediated by its receptor GHR. The study on Atlantic salmon [61] provides strong evidence that the expression of *GHR* regulates the production of IGF-I in fish, which has a pivotal role in growth determination [62]. Therefore, the *GHR* gene should be further examined as a possible candidate for growth improvement in finfish. *GHRH* codes for a peptide important in upregulating the *GH* expression [63]. Six signal nucleotide polymorphisms (SNPs) in the 5’UTR of cattle *GHRH* gene were revealed association with growth traits including weight improvement [64]. A SNP in the *GHRH* fourth intron out of Arctic charr was related to a significant increase (9.4%) in growth rate of early life stages [65]. However, there are few studies on the variability within this gene of flatfishes reported despite the importance of GHRH in the somatotropic hormonal axis.

Myostatin (MSTN, also known as GDF-8), a member of the transforming growth factor-β (TGF-β) superfamily, functions as a negative regulator of skeletal muscle development and growth [66]. Suppression of *MSTN* in the transgenic fishes resulted in the increase of muscle production [67]. Recently, *MSTN* has become a focal gene in the polymorphism detection and association studies towards selective breeding for growth traits in livestock [68] and some aquaculture species, such as the mollusk [69] and genetically improved farmed tilapia [70]. Myf-5 is a key member of the myogenic regulatory factors (MRFs) with a characteristic basic helix-loop-helix (bHLH) domain. A mutation of a single-base pair in one intron of the *myf5* gene associating with increases of the cattle body weight [71] suggests that this gene is a potential candidate for marker-assisted selection of economic varieties. The reported studies of *myf5* mainly focused on molecular structure, dynamic expression, and promoter analysis in some fish [72,73] excluding *S*. *maximus*. Reports on polymorphisms of this gene have not been found as yet.

Fatty acid-binding proteins (FABPs), belonging to a superfamily of intracellular lipid-binding proteins, occur ubiquitously in tissues of vertebrates and invertebrates with distinct expression patterns for the individual FABPs. These proteins have multiple proposed roles, such as promoting the cellular uptake and transfer of fatty acids (FAs), targeting FAs to specific metabolic pathways, and involving in the regulation of gene expression and cell growth [74]. There are various members of the FABP family, of which liver (L-), intestinal (I-) and heart (H-) are the dominating types [75]. As shown in Table 3, transcripts of the three FABPs were all detected in the present transcriptome. To date, the progress of fish *fabp*s is limited to the expression patterns in Atlantic salmon [76] and the promoter function in zebrafish [77]. Consequently, there exists a broad area of interest to explore the functions of FABP family in turbot *S*. *maximus*. SNP in the gene encoding 5-hydroxytryptamine receptor has shown the significant association with growth trait in crustaceans [78], while its functions and polymorphisms in fish are hardly ever to be investigated.

Prolactin (PRL) is an important regulator with multiple biological functions through binding to its receptor (PRLR) in fish [79]. Studies of calcium uptake in larval tilapia [80] indicate that PRL could be involved in regulating calcium balance as has been suggested in adult fish, while more evidence needs to be revealed about the role of PRL in larval calcium balance, because calcium accretion is important for numerous processes, particularly skeletal formation a process initiating soon after hatch in fish. Few reports in fish support a somatotropic action for PRL. It was suggested to influence growth of Mozambique tilapia with stimulating liver IGF-I production [81]. Additionally, transcripts of PRL and PRLR have been identified in association with post-hatching development of fish larvae [82]. Regarding the effect of PRL in fish immune function, it is increasingly clear that PRL is an important modulator mediated by PRLR [83], enhancing mitosis as well as phagocytic activity of leucocytes [84], and stimulating immunoglobulin production [85]. Immunoregulation in larval fish is crucial for commercial interest to be attained in aquaculture. Overall, PRL exerts multiple functions with receptor PRLR in fish, and probably also in fish larvae, and may have a different spectrum of activities in different species. Further work is required to fully characterise their activities.

It is well-reasoned that the different expression abundance of growth related genes are mainly responsible for the marked sex difference in growth rate of turbot. The expression of many selected genes in Table 3 was detected to be significantly downregulated in the male transcriptome, compared with that of the female, including gene of GH precursor, *igfbp2*, *myf5*, *fabp*, *htr*, *prl* and *prlb*, all of which have been proved to have positive effects on growth of vertebrates. The result may provide supplementary evidence on the validation of the chosen growth genes.

#### Candidate genes related to immune response

The innate and adaptive immunity composes the immune defense system of fish, and the former plays a major role in immune response than that of the latter in fish [86]. The knowledge of the relevant genes for immune response in *S*. *maximus* has greatly increased recently with the effort of high-throughput sequencing. Pereiro et al. [87] detected the antiviral transcripts of immune-related tissues from infected turbots using 454-pyrosequencing and provided a rich source of data to increase the knowledge of *S*. *maximus* immune transcriptome. Consequently, a large number of contigs and singletons involved in innate and acquired immunity were discovered after combining the Sanger and pyrosequencing data [15]. Here, a significant number of genes detected in our transcriptome (see S2 Table) were confirmed to be the main components of the immune pathways (complement, Toll-like receptor signaling, B cell receptor signaling, T cell receptor signaling and programmed cell death), agreed with the previous two results. Certainly, several interesting genes related to innate and acquired immunity were presented in Table 4.

**Table 4:** The intriguing immune-related genes identified in the turbot transcriptome.

	Gene Name	Annotation	Query Name
**Toll-like receptor signaling pathway**	*TLR1*	Toll-like receptor 1	comp32484_c3_seq1
	*TOLLIP*	Toll-interacting protein	comp21400_c0_seq1;comp23286_c0_seq1;comp23286_c1_seq1
	*TRAF3*	PREDICTED: TNF receptor-associated factor 3-like	comp22885_c1_seq1;comp23509_c0_seq1
	*TLR21*	Toll-like receptor 21	comp32484_c3_seq1
	*TLR22*	Toll-like receptor 22	comp36502_c11_seq1
	*IL-34*	Interleukin-34	comp30033_c0_seq1
	*IL18R1*	Interleukin-18 receptor 1	comp3046_c0_seq1;comp22532_c0_seq1
	*IL1R1*	Interleukin-1 receptor 1	comp28977_c1_seq1
**Apoptosis or programmed cell death**	*TRAF2*	PREDICTED: TNF receptor-associated factor 2-like	comp26413_c0_seq1;comp26467_c3_seq1;comp26467_c5_seq1
**complement pathway**	*C9*	PREDICTED: Complement component C9-like	comp37672_c0_seq1
**Other immune molecules**	*SLEP*	Selectin P	comp35827_c0_seq1
	*COLEC*	Collectin 10/11/12	comp31521_c1_seq1;comp31124_c0_seq1;comp30265_c0_seq1;comp31642_c0_seq1

Recognition of pathogen-associated molecular patterns (PAMPs) mediated by pattern-recognition receptors (PRRs) is critical to the initiation of innate immune responses. PRRs sense the conserved molecular structure (PAMPs) of a pathogen and induce subsequent host immunity through multiple signaling pathways for eradicating the pathogen [88]. Toll-like receptors (TLRs) are the first characterized PRRs, sharing structural and functional similarities from Drosophila to humans [89]. They are believed to play a crucial role in host defense of pathogenic microbes in innate immune system through recognizing PAMPs expressed on infectious agents. Three crucial members of *TLR* group (*TLR1*, *TLR21* and *TLR22*) were identified in the genomic database of turbot. TLR1 plays an essential role in pathogen recognition and activation of innate immunity [90]. In recent years, *TLR1* has been characterized in a number of fish, such as *Tetraodon nigroviridis* [91], *Epinephelus coioides* [92], and *P*. *olivaceus* [93]. TLR22 occurrs exclusively in aquatic animals with similar functions of mammalian TLR3, and supervises the infection of dsRNA virus to alert the immune system for antiviral protection in fish [94]. *TLR22* evolves functional diversification and adaptation of the response to different PAMPs while the information of *TRL21* is still rare in the model zebrafish [95], not to mention turbot. It is evident that TNF receptor-associated factor 3 (TRAF3) plays multiple roles in mammalian T and B lymphocytes [96], such as antagonizing the effects of TRAF2 in NF-κB activation, but the function of TRAF3 in the two piscine immune cells needs to be investigated. Interleukin-34 (IL-34) is a cytokine that promotes the differentiation and viability of monocytes and macrophages through the colony-stimulating factor-1 receptor [97]. Genes encoding two interleukin receptors essential for ILs mediated signal transduction were first detected in the turbot cDNA library. *C9* encodes the final component of the complement system, which participates in the formation of the membrane attack complex (MAC) that assembles on bacterial membranes to form a pore, permitting disruption of bacterial membrane organization. C-type lectin is a large family that includes all known collectins and selectins in animals. The collectins with carbohydrate recognition domains, are secreted proteins that play important roles in the innate immune system by binding to carbohydrate antigens on microorganisms, facilitating their recognition and removal. The selectin P may play a role in inflammatory response. The encoding genes of these C-type lectins found in the present database will be useful to investigate their roles in host defense of *S*. *maximus*. Many cytokines that play pivotal roles in the mammalian specific immunity have been validated in teleost fish, including transforming growth factor-β (TGF-β), and a number of IL involved in adaptive immune responses. IL-15 was discovered in relevance to the adaptive immune response of pufferfish [98] and rainbow trout [99]. All isoforms of the TGF-β family have been identified in a variety of teleosts, such as *Danio rerio* [100], *Oncorhynchus mykiss* [101], *Morone saxatilis* [102]. However, information on biological functions of these molecules in fish immune system remains limited. The identified nucleotide sequences of IL-15, receptors of other ILs, TGF-β and related receptors in this study will support the foundation to make their functions in turbot adaptive immunity clear.

### Molecular markers

#### SSR characterization and polymorphism evaluation

**Fig 6:**
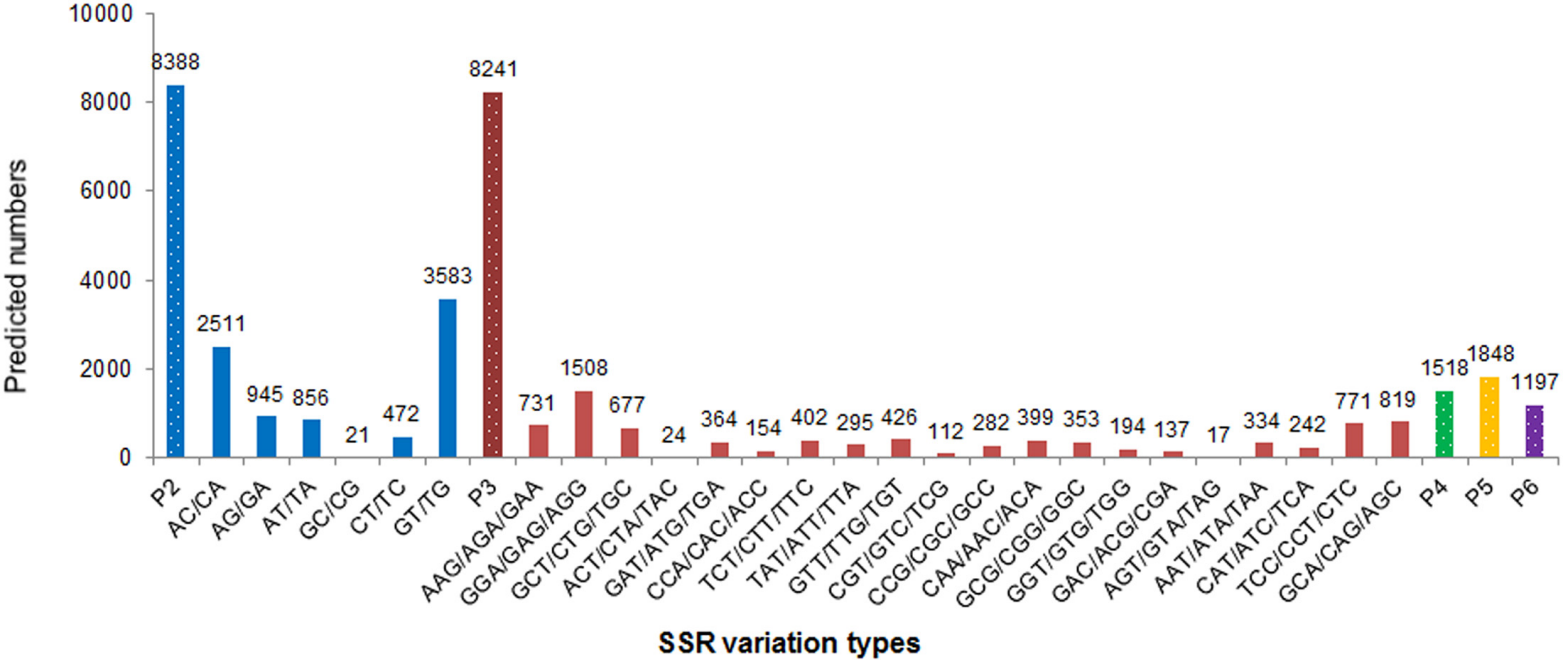
Frequency distribution of SSRs by motif length found in *Scophthalmus maximus*.

SSRs have been widely used in construction of genetic linkage, QTL analysis and assessment of genetic diversity in aquaculture species, due to the distinct advantages of high variability, abundance, neutrality and co-dominance [103]. An important emerging application of high-throughput Illumina-Solexa is the identification of molecular markers from genomic DNA. Our search revealed 21,192 SSRs were contained in ESTs of the transcriptomic dataset, of which 39.58% were di-nucleotide repeats, followed by 38.89% tri-nucleotide repeats and 21.53% tetra/penta/hexa-nucleotide-repeats (Fig 6). The most abundant SSR repeat types of animals are generally believed to be di-nucleotide repeats [36,41], and our findings are coincident with this conclusion. In the di-nucleotide repeats motifs, (GT/TG)n and (AC/CA)n were the two predominant types with frequencies of 42.72% and 29.94%, respectively. Among the 20 types of tri-nucleotide repeats, (GGA/GAG/AGG)n, (GCA/CAG/AGC)n, (TCC/CCT/CTC)n and (AAG/AGA/GAA)n were the leading types with a combined frequency of 46.46%.

**Fig 7:**
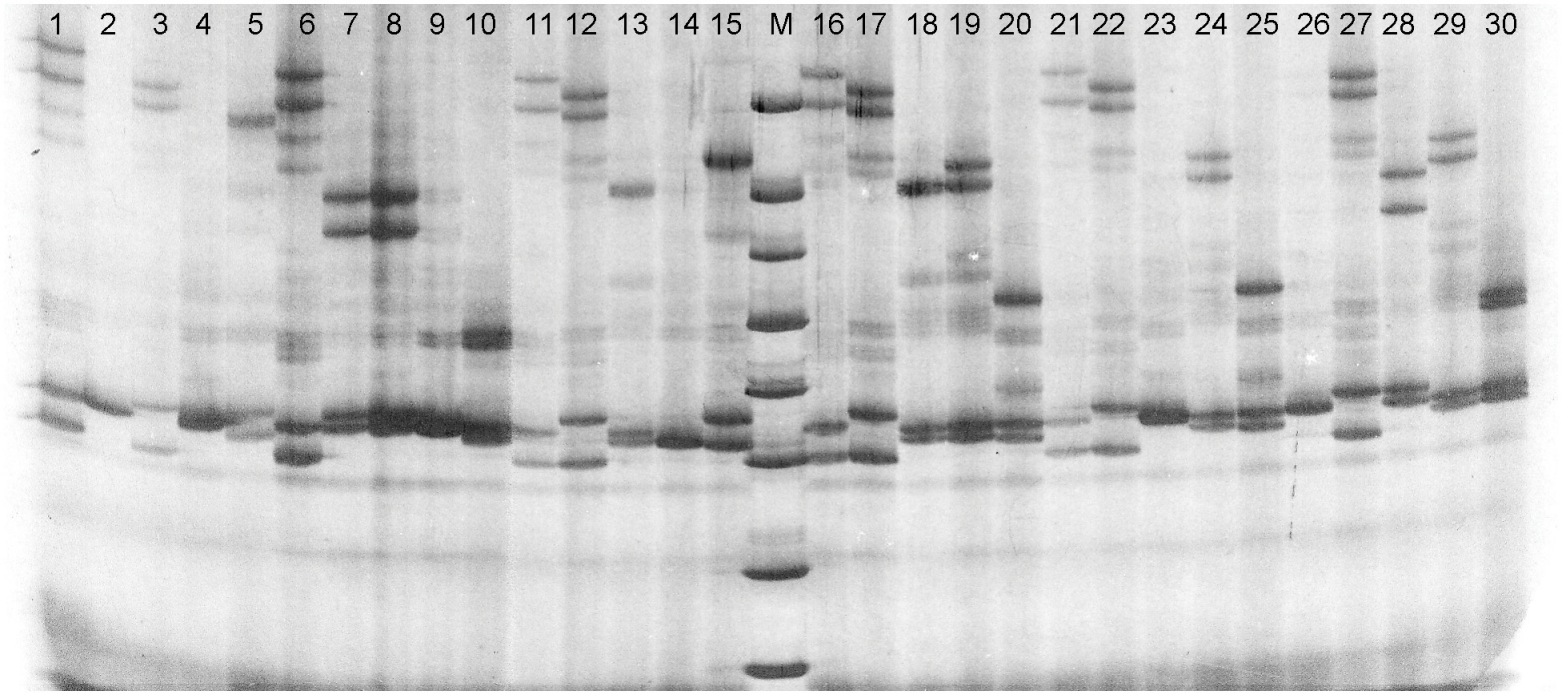
Polyacrylamide gel electrophoresis for one SSR marker (comp15993_c0_seq1) in the 30 individuals.

In this study, 2,357 (11.22%) SSR-containing sequences enabled the design of primers (S3 Table), which are the highly desirable development of SSRs for this species. One hundred SSRs were randomly selected for primer synthesis and identification, among which, 70 received clearly amplified target products in PCR amplification. Of these, seventeen were available for the further genetic linkage map construction based on a turbot family through polymorphism validation in a population of 30 individuals (Fig 7). Using the 17 primer pairs, we described the genetic structure characterization of a turbot family including 90 progenies (Table 5). A total of 39 alleles were identified, with an average of 2.29 alleles per locus. The polymorphic information content (*PIC*) ranged from 0.22 to 0.7 with an average of 0.35, indicating that these identified EST-SSRs were at least moderate polymorphic.

**Table 5:** Characterization of 17 polymorphic SSR loci in a turbot family of 90 individuals.

Transcripts ID	Repeat motif	Primer sequences (5’-3’)	Length	*Ta*	*Na*	*He*	*Ho*	*PIC*
**comp15993_c0_seq1**	(GACCTG)3	F: ACTGGAGACGGTCCGATACCTG	129	58	5	0.75	0.79	0.65
		R: ACTCATGGTGCTGGTCCAGGTC						
**comp18360_c0_seq1**	(CCTGGA)3	F: ACTCTCCGCCGCTCAGTAAGG	125	58	2	0.5	0.39	0.27
		R: GTCTCCGTCGTCCAGGTCGTC						
**comp20204_c0_seq1**	(CTC)6	F: GTGAGCTGGTGCTGAGTGTAGG	132	57	2	0.5	0.41	0.27
		R: GAGGATGAGGCGGAGGAAGAG						
**comp20308_c0_seq1**	(GAC)5	F: CATGCAGCAGGAGACCAGGAG	233	57	2	0.5	058	0.33
		R: CGTCGTCGTCGTCGTCATCATC						
**comp20617_c0_seq1**	(GGA)5	F: CCTTCACCCTTTCACCCTTCGC	120	57	2	0.5	0.35	0.37
		R: TATCCACCACAGCCAGCACCTC						
**comp21529_c1_seq1**	(GCT)6	F: TTCTTTTCCCCACGGGGCTTTG	120	58	2	0.5	0.43	0.28
		R: GCCGGTTGGATCAGAGCTGTTC						
**comp21594_c1_seq1**	(CGC)6	F: CTCCGCCACACGCAGAAATCTC	213	58	2	0. 5	0.53	0.31
		R: TCGCACCTGTCTCCCATCGTC						
**comp29608_c1_seq1**	(GCTA)5	F: AGACCAGGATCCGAGTCGAACC	148	57	2	0.5	0.48	0.37
		R: TCAGGTGGCTTCGCGTTTACAG						
**comp30754_c0_seq1**	(AGGTTC)6	F: CAACCTCCTCGCTGCTGCTTC	135	59	2	0.5	0.42	0.28
		R: AGGAACCGATGGAGGCGTCTG						
**comp31253_c0_seq1**	(TGC)6	F: GATCGGCCCATCGTTTGGACTG	143	58	2	0.5	0.46	0.29
		R: TCCCAGCACCACAGCCTCAAG						
**comp31355_c0_seq1**	(AAT)7	F: ACGACCAACTCCAACTCACAGC	178	58	2	0.5	0.35	0.25
		R: TGGCAGGCAGCAACAAGACAC						
**comp34582_c0_seq1**	(CGTG)5	F: GTTCTGCTGCACCCTGCTTCC	187	59	2	0.5	0.53	0.31
		R: GCGACGACTTGCTCACACAAAC						
**comp36059_c15_seq1**	(CGTG)6	F: AAGGAGGCGGTGAACCAGGTC	207	58	2	0.75	0.64	0.5
		R: GCGACGTGGACGAGAGTGAAG						
**comp36274_c2_seq1**	(TGG)7	F: GTCCTGGTGCTCTGACAGAAGC	126	58	2	0.5	0.49	0.3
		R: ACTCTGGACCACCACCACCAC						
**comp36960_c0_seq1**	(AG)9	F: GTCCGTGTGTTCGCCAGACATG	123	58	2	0.5	0.51	0.3
		R: TGACTTCCTGACGCCCGCTTC						
**comp37121_c6_seq1**	(GAG)6	F: GGAGACGAACGGGAGTCAAAC	174	58	2	0.5	0.29	0.22
		R: CTGGTTCCTGATGCCGCACAG						
**comp37181_c7_seq1**	(GCTC)5	F: AAGAGGCCGGTGTCAGGACTG	182	58	4	1	1	0.7
		R: GTGAGCCCTTCTCCCGTCAAAC						

*Ta* annealing temperature, *He* expected heterozygosity, *Ho* observed heterozygosity, *Na* observed number of alleles, *PIC* polymorphic information content.

#### SNP characterization and validation

**Fig 8:**
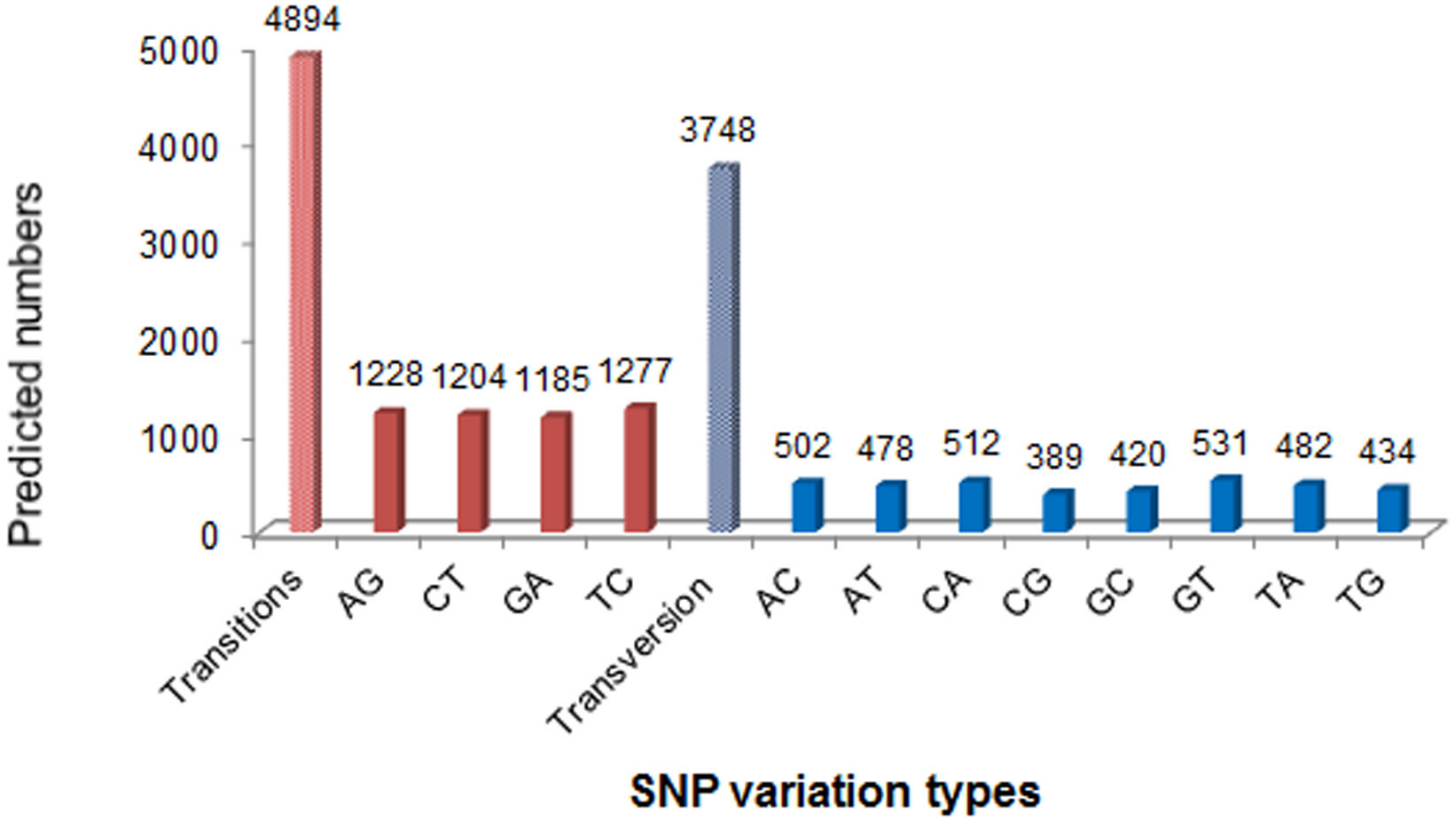
Distribution of putative single nucleotide polymorphisms (SNPs) containing in *Scophthalmus maximus* ESTs.

Putative SNPs were detected from alignments of multiple sequences during contig assembly. In this study, a total of 8,642 SNPs were obtained, of which 4,894 were transitions (Ts) and 3,748 were transversions (Tv), giving a mean Ts: Tv ratio of 1.306:1 across the turbot transcriptome (Fig 8). The four transitions were the most common SNP types, and GC/CG transversions were the least SNP types on account of the difference in base structure and number of hydrogen bonds between different base [23,35].

**Fig 9:**
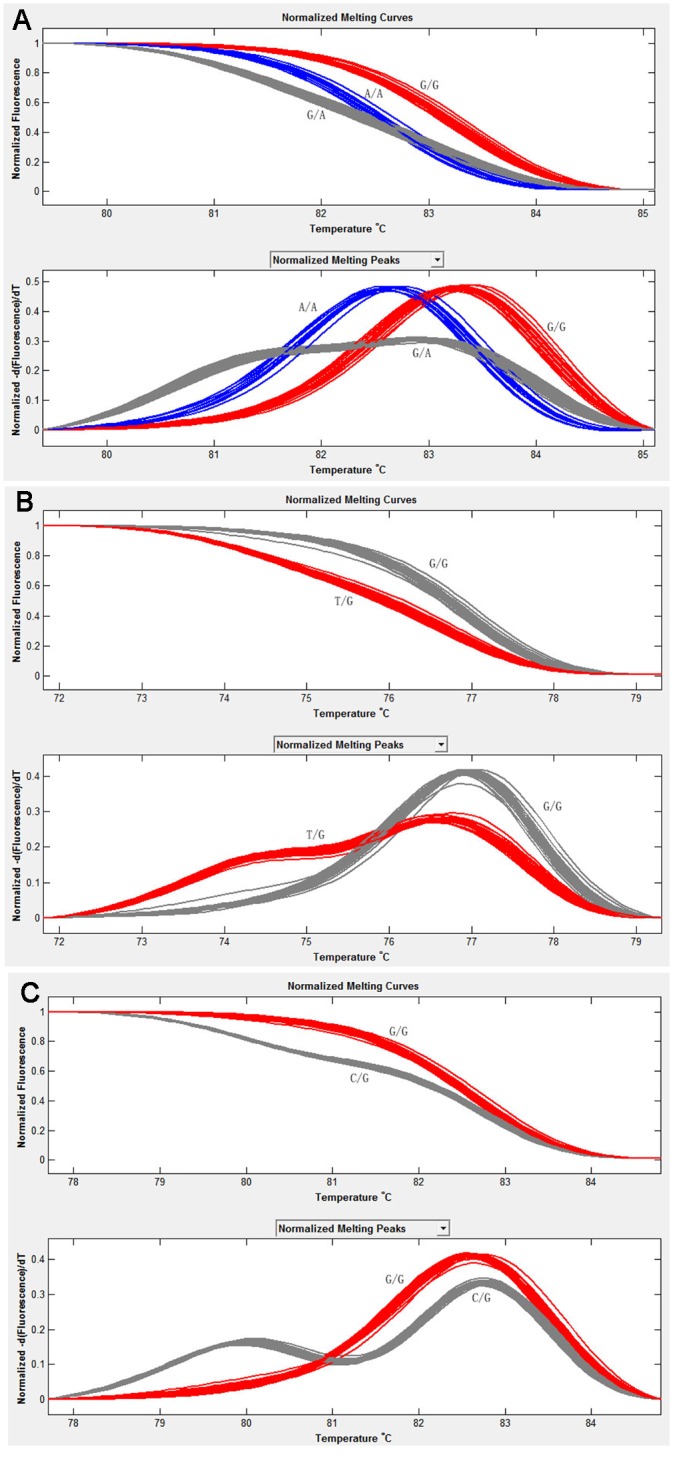
Genotyping result using HRM with small amplicon. (A) S10 genotyping; (B) S11 genotyping; (C) S14 genotyping.

To verify the potential SNPs, 63 primer pairs were designed according to 45 contigs containing SNP with high coverage, of which 21 pairs amplified the exclusive products through 2% agarose gel electrophoresis detection. The distinctly different genotypes on the twenty-one SNPs were validated by HRM genotyping of 96 samples (Fig 9). The polymorphism evaluation indicated that most SNPs (19 of 21) are moderate polymorphic sites and matched to Hardy-Wenberg equilibrium (S4 Table). Moreover, the effective annotations of genes with the identified SNP locations showed that these genes are involved in regulation of cell cycle, cytoskeleton, energy transformation and RNA processing [104].

Taken together, the results obtained in this study indicated that these potential molecular markers identified within the ESTs will enable more detailed studies on evolutionary genomics, comparative mapping, and QTL analysis of *Scophthalmus maximus*.

## Conclusion

Here we report the comprehensive transcriptome of major tissues in turbot *Scophthalmus maximus*, a commercially important flatfish in China. The large amount of generated sequences (71,107 putative transcripts) will enrich enable genomic resources in turbot and therefore to improve available sequence databases for gene discovery. A significant number of putative genes related to economic traits were identified to facilitate genomics approaches for controlling sex ratio, improving growth performance and resistance to pathogens in domesticated stocks used for aquaculture. A large amount of genetic markers was detected, providing new tools for genomic studies and management of molecular assistant selection in cultured populations.

## Supporting Information

S1 File All assembled sequences of contigs. (RAR)

S1 Table The detailed annotation information of genes. (XLS)

S2 Table All identified genes related to immune response. (XLS)

S3 Table Summary of EST-SSRs with 2~6 bp motif repeats and primers. (XLS)

S4 Table Genetic variability at 21 SNPs in *Scophthatmus maximus*. (DOC)
