# Supplementary material for: Transcriptome Analysis for Identification of Genes Related to Gonad Differentiation, Growth, Immune Response and Marker Discovery in The Turbot (Scophthalmus maximus)
Source: PLoS One. 2016 Feb 29;11(2):e0149414. doi: 10.1371/journal.pone.0149414 (PMC4771204; doi:10.1371/journal.pone.0149414)
Supplement: S4 Table — (DOC) [file pone.0149414.s005.doc]

| Locus name | *PIC* | *N*_e_ | *H*_o_ | *H*_e_ | MAF |
| --- | --- | --- | --- | --- | --- |
| 8S82 | 0.370 | 1.958 | 0.854 | 0.495 | 0.4271 |
| CS1 | 0.373 | 1.986 | 0.915 | 0.502 | 0.4574 |
| CS10 | 0.373 | 1.983 | 0.907 | 0.502 | 0.4535 |
| CS3 | 0.374 | 1.958 | 0.505 | 0.495 | 0.4271 |
| CS4 | 0.374 | 1.992 | 0.723 | 0.503 | 0.4681 |
| CS5 | 0.235 | 1.374 | 0.325 | 0.276 | 0.1625 |
| CS6 | 0.259 | 1.441 | 0.333 | 0.310 | 0.1889 |
| CS9 | 0.374 | 1.991 | 0.841 | 0.503 | 0.4659 |
| S10 | 0.371 | 1.968 | 0.447 | 0.497 | 0.4362 |
| S11 | 0.446 | 2.051 | 0.638 | 0.518 | 0.0426 |
| S12 | 0.375 | 2.000 | 1.000 | 0.505 | 0.5000 |
| S14 | 0.308 | 1.614 | 0.511 | 0.384 | 0.2553 |
| S15 | 0.358 | 1.878 | 0.575 | 0.472 | 0.3723 |
| S16 | 0.374 | 1.990 | 0.605 | 0.503 | 0.4651 |
| S22 | 0.344 | 1.792 | 0.447 | 0.447 | 0.3298 |
| S3 | 0.305 | 1.600 | 0.417 | 0.379 | 0.2500 |
| S42 | 0.332 | 1.724 | 0.333 | 0.425 | 0.3000 |
| S62 | 0.366 | 1.932 | 0.479 | 0.488 | 0.4062 |
| S63 | 0.375 | 1.999 | 0.896 | 0.505 | 0.4896 |
| S7 | 0.226 | 1.352 | 0.256 | 0.264 | 0.1538 |
| S9 | 0.374 | 1.992 | 0.648 | 0.503 | 0.4688 |
